# Continuum finite element analysis generalizes *in vivo* trabecular bone microstructural strength measures between two CT scanners with different image resolution

**DOI:** 10.1088/2057-1976/acbb0a

**Published:** 2023-02-22

**Authors:** Indranil Guha, Xiaoliu Zhang, Syed Ahmed Nadeem, Steven M Levy, Punam K Saha

**Affiliations:** 1 Department of Electrical and Computer Engineering, College of Engineering, University of Iowa, Iowa City, IA, United States of America; 2 Department of Radiology, Carver College of Medicine, University of Iowa, Iowa City, IA, United States of America; 3 Department of Epidemiology, College of Public Health, University of Iowa, Iowa City, IA, United States of America; 4 Department of Preventive and Community Dentistry, College of Dentistry, University of Iowa, Iowa City, IA, United States of America

**Keywords:** osteoporosis, trabecular bone, micro-structure, nonlinear FE modelling, bone strength, multiscanner generalizability

## Abstract

Fragility of trabecular bone (Tb) microstructure is increased in osteoporosis, which is associated with rapid bone loss and enhanced fracture-risk. Accurate assessment of Tb strength using *in vivo* imaging available in clinical settings will be significant for management of osteoporosis and understanding its pathogenesis. Emerging CT technology, featured with high image resolution, fast scan-speed, and wide clinical access, is a promising alternative for *in vivo* Tb imaging. However, variation in image resolution among different CT scanners pose a major hurdle in CT-based bone studies. This paper presents nonlinear continuum finite element (FE) methods for computation of Tb strength from *in vivo* CT imaging and evaluates their generalizability between two scanners with different image resolution. Continuum FE-based measures of Tb strength under different loading conditions were found to be highly reproducible (ICC ≥ 0.93) using ankle images of twenty healthy volunteers acquired on low- and high-resolution CT scanners 44.6 ± 2.7 days apart. FE stress propagation was mostly confined to Tb micro-network (2.3 ± 1.7 MPa) with nominal leakages over the marrow space (0.4 ± 0.5 MPa) complying with the fundamental principle of mechanics at *in vivo* imaging. In summary, nonlinear continuum FE-based Tb strength measures are reproducible among different CT scanners and suitable for multi-site longitudinal human studies.

## 1. Introduction

Osteoporosis is an age-related degenerative bone disease characterized by reduced bone mineral density (BMD) and associated with increased fracture-risk (Watts *et al* 2010). One in every four men and every two women suffers at least one osteoporotic fragility fracture in their lifetime (National Osteoporosis Foundation 2017). Dual-energy x-ray absorptiometry (DXA)-measured areal BMD is clinically used to detect osteoporosis (Seeman and Delmas 2006). However, BMD explains only 60%–70% of the bone’s mechanical competence (Wehrli *et al* 2002), and the remainder is contributed by various factors including trabecular bone (Tb) microstructural basis (Parfitt *et al* 1983, Kleerekoper *et al* 1985, Legrand *et al* 2000, Van Ruijven *et al* 2007, Boutroy *et al* 2008, Liu *et al* 2010). Different Tb microstructural measures (Bouxsein *et al* 2010, Chen *et al* 2018) have been widely investigated with the overall goal being able to predict Tb strength, fragility, and fracture-risk.

Finite element (FE) analysis (FEA) computationally simulates a mechanical loading condition on a target object, and it has been popularly applied to compute bone strength. FEA has been applied to assess femoral strength using CT (Keyak *et al* 2005, Keyak *et al* 2013, Rayudu *et al* 2020). However, these methods were implemented using significantly larger mesh elements compared to the average human Tb thickness and were primarily aimed at deriving mechanical properties based on bone geometry and macro-distribution of bone density. Early applications of FEA to compute bone microstructural strength were based on micro-CT imaging of Tb bone specimens (Ulrich *et al* 1998, Chevalier *et al* 2007, Van Ruijven *et al* 2007, Rajapakse *et al* 2010). It has been observed that micro-CT derived FEA measures closely agree with mechanical experimental finding, and, often, micro-CT derived FEA measures are considered as the gold standard. FEA was later adopted for high-resolution (HR) peripheral quantitative computed tomography (HR-pQCT) (MacNeil and Boyd 2007, Nishiyama *et al* 2013) and magnetic resonance imaging (MRI) (Rajapakse *et al* 2010, Zhang *et al* 2013) for *in vivo* assessment of Tb microstructural strength. Previously reported HR-pQCT and MRI-based FEA methods required segmentation and separation between the marrow space and Tb micro-network.

Recently, emerging whole-body CT scanners have drawn interest as an alternative to HR-pQCT and MRI for *in vivo* bone micro-imaging at peripheral sites due to their widespread availability in clinical setting, high-resolution, fast scan-speed, and ultra-low radiation (Chen *et al* 2018). In an *ex vivo* study, (Bauer *et al* 2014) computed failure loads of small cadaveric Tb specimens using CT-based FEA, where bone voxels were separated from marrow by thresholding and a constant Young’s modulus was used for bone voxels in an oriented brick mesh model (Guha *et al* 2022) established a nonlinear continuum FEA method for *in vivo* ankle CT scans that computes Tb microstructural strength without requiring separation of Tb micro-network from marrow space, while capturing the impacts of micro-distribution of bone mineral on strength. Relaxation of the segmentation of Tb microstructure by Guha *et al*’s method allows FEA-based study of bone strength at relatively low-resolution (LR) imaging frequently available in routine clinical scans, while accounting for bone micro-distribution at the level of Tb network. A major challenge with CT-based FEA methods emerges due to the wide variation in spatial resolution and other imaging and reconstruction features among different CT scanners along with rapid upgrades in technology causing inconsistency in Tb micro-morphometric measures in multi-site and longitudinal bone studies (Chen *et al* 2018). Although, the paper by (Guha *et al* 2022) demonstrated that their method offers reliable measures of Tb microstructural strength at *in vivo* peripheral CT imaging, variability of their measures derived from muti-scanner CT images with varying spatial resolution has not yet been established. This paper is aimed to examine the multi-scanner variability of Guha *et al*’s Tb FEA measures in an *in vivo* study. Specifically, we evaluate the reproducibility of Tb FEA measures derived from *in vivo* ankle CT scans of human participants on low- and high-resolution clinical CT scanners.

## 2. Methodology

In this section, we describe the CT-based nonlinear continuum FEA for computing Tb Young’s modulus, as well as the experimental plans to examine the reproducibility of the method.

### 2.1. Human subjects and *in vivo* imaging

Twenty healthy volunteers (age (mean±standard deviation (std)): 26.2 ± 4.5 years; 11 males and 9 females) were recruited, and distal tibias of their left legs were scanned on both LR Siemens FLASH and HR Siemens FORCE scanners at an interval of 44.6 ± 2.7 days. This study was approved by The University of Iowa Institutional Review Board (IRB ID # 201505701), and written informed consents were obtained from participants. This study was conducted in accordance with the Declaration of Helsinki.

#### 2.1.1. Siemens FLASH CT imaging

Spiral CT scans were acquired using single x-ray source at 120 kV and 200 effective mAs with 1 sec rotation speed and pitch factor of 1.0. Total effective dose was equivalent to 170 *μ*Sv ≈ 20 days of environmental radiation. Vendor-provided special U70u kernel was used to reconstruct CT images at 200 *μ*m slice spacing and 176 *μ*m pixel-size.

#### 2.1.2. Siemens FORCE CT imaging

Spiral CT scans were acquired using single x-ray source at 120 kV and 100 effective mAs with 1 sec rotation speed and pitch factor of 1.0. Total effective dose equivalent was 50 *μ*Sv ≈ 5 days of environmental radiation. Vendor-provided special Ur77u kernel with Edge Technology was used to reconstruct CT images at 200 *μ*m slice-spacing and 176 *μ*m pixel-size.

For both scanners, Siemens z-UHR scan mode was applied, enabling Siemens double z-sampling technology achieving high structural resolution. The Siemens FORCE scanner used Stellar^Infinity^ detectors with anti-scatter collimator, which improved signal detectability and reduced noise producing similar image quality and signal-to-noise ratio as LR scans at a reduced radiation dose. Therefore, lower radiation dose was used for the Siemens FORCE scanner. A Gammex RMI 467 Tissue Characterization Phantom (Gammex RMI, Middleton, WI) was scanned on LR and HR scanners using the matching CT imaging and reconstruction protocol to calibrate CT Hounsfield numbers (HU) into BMD (mg/cc) values.

### 2.2. Image pre-processing

Image processing was applied to each CT image to — (1) align the tibial bone axes with the image coordinate axes and extract volume of interest (VOI) and (2) compute the calcium hydroxyapatite (CHA) density (g/cc) using the calibration phantom scan.

#### 2.2.1. Bone alignment and voi selection

**Figure 1.**
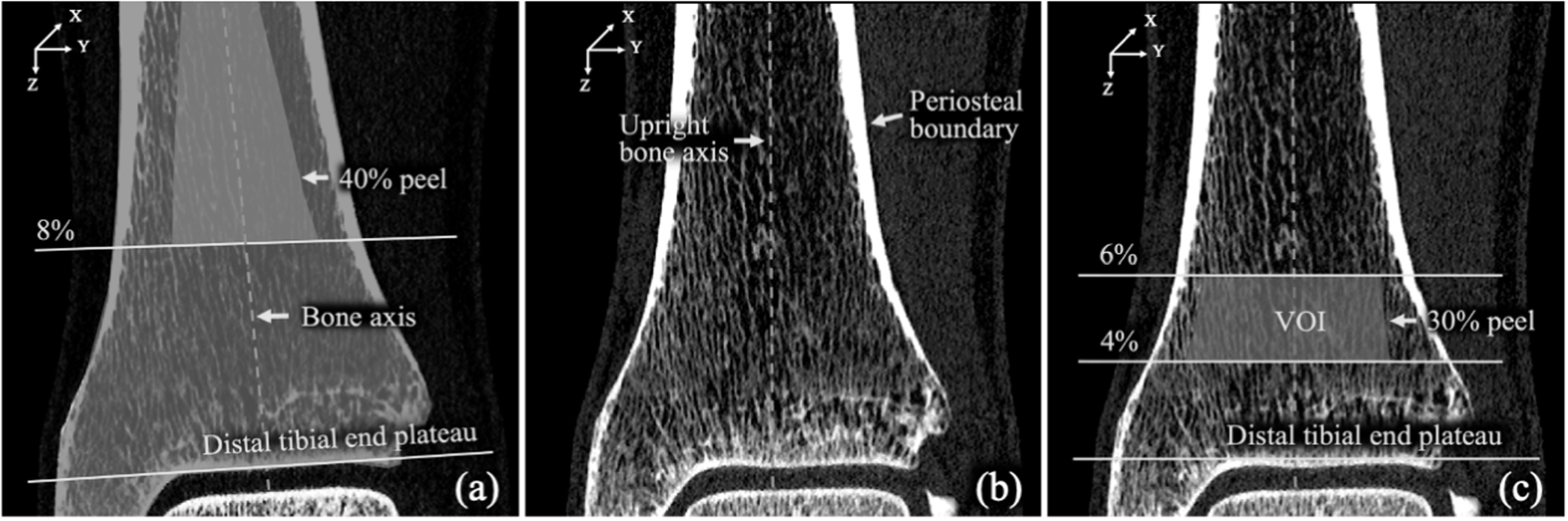
Bone alignment and VOI selection on a distal tibia CT scan for FEA. (a) Segmented tibial bone region (green) on a sagittal image slice and quasi-cylindrical region (magenta) for bone axis computation. (b) A sagittal CT slice after alignment of the tibial bone axis. (c) Selected VOI (blue) for FEA.

The bone alignment and VOI selection process is described in figure 1. First, the tibial bone was segmented from each CT scan using a previously validated algorithm (Li *et al* 2015). Forty percent in-plane peeling was applied to the tibial bone, and the peeled bone proximal to the 8% tibial site from the reference distal end plateau was used to compute the tibial bone axis; see (a). Subsequently, the tibial bone axis was aligned with the image *z*-axis (see (b)), while the *x*-axis was aligned with the tibiofibular direction. Simultaneously, the image was interpolated at 150 *μ*m isotropic voxel resolution using the windowed sync method (Meijering *et al* 1999). These transformations were combined to reduce the image resolution loss during interpolation. The bone alignment was necessary to align longitudinal trabeculae with the compressive loading direction. An axial VOI was selected in the aligned bone covering 4%–6% tibial length (∼6.75 mm) after 30% peel from the periosteal boundary (see (c)). Tibial bone segmentation and alignment and selection of axial VOI were independently applied to the LR and HR CT scans without registration to assess the generalizability of the entire processing cascade.

#### 2.2.2. CHA and Ash density computation

CT numbers in HU were calibrated into BMD values using CT scans of a calibration phantom; see section 2.1. Next, the BMD value ${\rho }_{{\rm{BMD}}}(p)$ at a voxel $p$ was converted to CHA density ${\rho }_{{\rm{CHA}}}\left(p\right)$ as follows:

\begin{eqnarray*}{\rho }_{{\rm{CHA}}}\left(p\right)=\displaystyle \frac{\left({\rho }_{{\rm{BMD}}}\left(p\right)-{D}_{{\rm{water}}}\right)}{({D}_{{\rm{CHA}}}-{D}_{{\rm{water}}})}\times {D}_{{\rm{CHA}}},\end{eqnarray*}

where, ${D}_{{\rm{CHA}}}\,$ (3.18 g cc^−1^) and ${D}_{{\rm{water}}}\,$ (1 g cc^−1^) are the known densities of CHA and water, respectively. Finally, the CHA density ${\rho }_{{\rm{CHA}}}\left(p\right)$ was converted to ash density ${\rho }_{{\rm{ash}}}\left(p\right)$ as follows:

\begin{eqnarray*}{\rho }_{{\rm{ash}}}\left(p\right)=0.0633+0.887{\rho }_{{\rm{CHA}}}\left(p\right)\end{eqnarray*}

### 2.3. Nonlinear continuum FE modelling and analysis

Nonlinear FEA was applied to a cubic mesh directly derived from the isotropic voxel grid within the upright quasi-cylindrical VOIs, where each voxel was represented by a cubic mesh element centred at its coordinate location. Each cubic element was represented by eight vertex and twelve edge elements to simulate the voxel geometry. Common edges and vertices of neighbouring mesh elements were used to specify their connectivity. The nonlinear isotropic material properties of each mesh element were derived from the ash-density of the corresponding image voxel.

**Figure 2.**
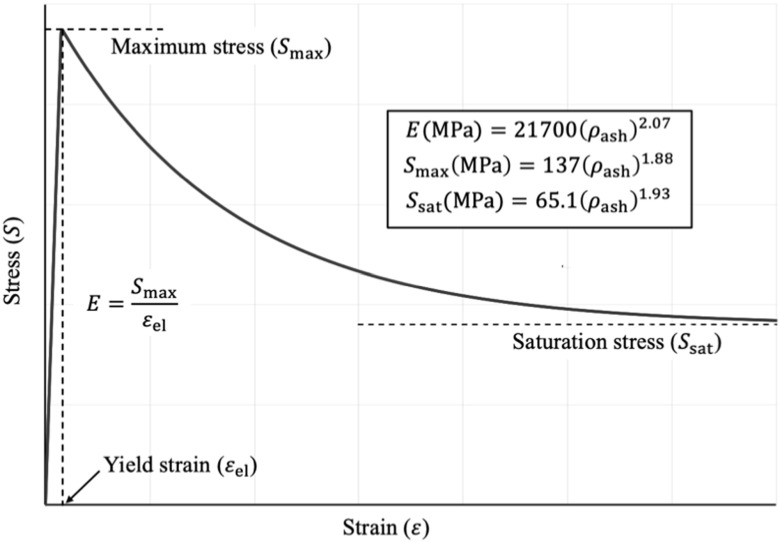
Nonlinear stress-strain relationship used to model bone material properties of voxel mesh elements.

We use ${p}_{{\rm{e}}}$ to denote the mesh element corresponding to the image voxel $p.$ The material behavior of the ${p}_{{\rm{e}}}$ was modelled using a nonlinear stress-strain relationship (figure 2) similar to (Keyak *et al* 1996). Elastic modulus $E\left({p}_{{\rm{e}}}\right)$ (MPa), maximum stress ${S}_{{\rm{\max }}}\left({p}_{{\rm{e}}}\right)$ (MPa), and saturation stress ${S}_{{\rm{sat}}}({p}_{{\rm{e}}})$ (MPa) at ${p}_{{\rm{e}}}$ were defined from ${\rho }_{{\rm{ash}}}(p)$ using the equations stated in figure 2. The mixed Swift-Voce isotropic hardening equation (Jang *et al* 2020) was used to define the stress-strain relationship ${S}_{{p}_{{\rm{e}}}}\left(\varepsilon \right)$|$\varepsilon \gt {\varepsilon }_{{\rm{el}}}\left({p}_{{\rm{e}}}\right)$ in the plastic region as follows:

\begin{eqnarray*}\begin{array}{c}{S}_{{p}_{{\rm{e}}}}\left(\varepsilon \right)={S}_{{\rm{\max }}}\left({p}_{{\rm{e}}}\right)+{R}_{1}* \left(1-{e}^{-b\left(\varepsilon -{\varepsilon }_{{\rm{el}}}\left({p}_{{\rm{e}}}\right)\right)}\right)\\ \,+{R}_{2}* \left(\varepsilon -{\varepsilon }_{{\rm{el}}}\left({p}_{{\rm{e}}}\right)\right)\,| \,\varepsilon \gt {\varepsilon }_{{\rm{el}}}\left({p}_{{\rm{e}}}\right)\end{array}\end{eqnarray*}

The parameter values of ${R}_{1}={S}_{{\rm{sat}}}\left({p}_{{\rm{e}}}\right)-{S}_{{\rm{\max }}}\left({p}_{{\rm{e}}}\right);$ ${R}_{2}=0;$ and $b=10$ were selected to resemble with Keyak *et al*’s stress-strain relationship. The softening behavior within the plastic phase was simulated using a negative value for ${R}_{1}.$ For FEA, we used a Poisson’s ratio of 0.3 and imposed boundary conditions to simulate experimental mechanical testing. Specifically, the mesh elements on the bottom surface were fixed in all directions, while a predefined displacement was applied on each mesh node on the top surface. For axial compressive FEA, the displacement was applied along the z-direction, while it was applied along x- (or y-) axis to simulate x- (or y-) shear loading conditions. Movements of top surface nodes along the directions orthogonal to the applied displacement were restricted. Displacements were gradually and uniformly applied in 50 sub-steps to achieve a total strain of 0.1%. After each sub-step, the average von Mises stress (MPa) (Logan 2011) over volume mesh elements on the top surface was computed and the stress-strain value was plotted. Finally, the slope of the linear section of the stress-strain curve was computed to determine the Tb Young’s modulus or simply modulus.

ANSYS (ANSYS Mechanical 2019 R2, Ansys Inc., Southpointe, Pennsylvania, USA) software tool was used to implement our nonlinear FEA algorithm. Cubic mesh elements were modelled using the ANSYS element type: 3-D 8-Node structural solid (SOLID185). ANSYS default values of 0.5% and 5% were used for force and displacement tolerance parameters, respectively. ANSYS NLGEOM mode was adopted, and Newton-Raphson’s method was applied for convergence.

### 2.4. Experiments and data analysis

Experiments were designed to (1) evaluate the reproducibility of the FEA derived modulus values under different loading conditions; and (2) examine the fundamental principle of structural mechanics at *in vivo* CT imaging that stress in continuum FEA propagates through Tb microstructure with nominal leakage into the marrow space. Descriptive statistics of means and standard deviation (std) of compressive and shear modulus values for LR and HR CT images were computed, and their linear correlation (r-value) and reproducibility in terms of intra-class correlation coefficient (ICC) were examined. Also, mean and std of different Tb microstructural measures (table 1) from LR and HR CT scans were computed, and their linear correlation and ICC values were examined to facilitate the discussions related to reproducibility of Tb microstructure and modulus measures between LR and HR CT scanners. For the purpose of evaluation in the second experiment, a previously validated algorithm (Chen *et al* 2016) was used for segmentation of Tb microstructure and marrow regions. Mean and std of stress over the Tb and marrow region were computed for each loading condition, and an unpaired t-test was performed to determine the significance in the difference between stress values over the two regions.

**Table 1.** List of CT-derived Tb measures examined in this paper.

Tb measure (unit)	Description
Tb.vBMD (g/cc)	Volumetric Tb mineral density
Tb.NA (1/mm)	Average area of Tb micro-network per unit VOI
Tb.PW (*μ*m)	Mean Tb plate width computed by VTA (Saha *et al* 2010).
Tb.Th (*μ*m)	Mean Tb thickness computed using star-line and fuzzy distance analysis (Liu *et al* 2014).
Tb.Sp (*μ*m)	Mean Tb spacing, i.e. the space between trabecular microstructures computed using star-line and fuzzy distance analysis (Liu *et al* 2014).

VTA: Volumetric topological analysis.

FEA experiments were executed on a Linux machine with 4 × 32 GB Tesla V100-SXM2 GPUs, 64 GB RAM, and 72 × 2.60 GHz Intel(R) Xeon(R) Gold 6240 CPUs. On an average, the Tb VOIs included approximately 277K and 91K edge and volume mesh elements, respectively. The mean±std of runtime of nonlinear FEA for all experiments was 75.1 ± 5.5 min.

## 3. Results and discussion

Mean and std of different modulus values computed from LR and HR CT images, and their linear and intra-class correlation coefficients are shown in table 2. High linear correlation (r ∈ [0.94 0.96]) was observed between different modulus values from LR and HR scans. Also, high ICC values (ICC ∈ [0.93 0.94]) were observed between the modulus values, demonstrating the reproducibility of the comprehensive analytic process including bone filling, bone alignment, VOI selection, and FEA applied independently on CT images at two different resolutions.

**Table 2.** Results of summary statistics (mean±std) and linear and intra-class correlation (ICC) analysis of different FEA-derived moduli from low- and high-resolution CT imaging.

Modulus (unit)	LR	HR	Pearson correlation (r)	ICC
Compressive	1622.1 ± 341.7	1698.9 ± 388.9	0.96	0.94
X-shear (MPa)	4050.1 ± 1156.3	4059.8 ± 1233.0	0.94	0.94
Y-shear (MPa)	3653.5 ± 825.7	3650.3 ± 914.6	0.94	0.93

Comparative results of different Tb measures computed from LR and HR CT scans are presented in table 3. It can be seen that, although high linear correlation (r ∈ [0.87 0.99]) was observed between the Tb measures derived from LR and HR scans, the reproducibility of these measures, except Tb.vBMD (ICC = 0.93), was low to moderate (ICC ∈ [0.39 0.85]). As BMD explains only 60%–70% of bone’s mechanical competence, high reproducibility of BMD values alone is not sufficient for assessment of the reproducibility of overall Tb microstructural strength from different CT scans at different resolutions. The nonlinear continuum FE modelling accounts for both BMD, as well as Tb microstructural distribution, and high reproducibility of the modulus measures demonstrates the potential of FEA as a generalizable tool for assessing Tb strength in multi-site and longitudinal studies using different scanners. Further, it is observed in table 3 that the Tb.Th metric has high linear correlation, but an ICC value noticeably smaller than the other four metrics. Tb.Th values (mean±std: 306.03 ± 44.52 *μ*m) derived using LR scans were higher compared to the values obtained from HR scans (237.27 ± 35.08 *μ*m). Observed high values of Tb.Th using LR scans may be attributed to greater partial voxel voluming and loss of thinner trabeculae in LR scans. This artefactual increase in Tb.Th values for LR scans contributed to small between-scanner ICC as compared to other four metrics.

**Table 3.** Results of summary statistics (mean±std) and linear and intra-class correlation (ICC) analysis of different Tb microstructural measures from low- and high-resolution CT imaging.

Tb measure (unit)	LR	HR	Pearson correlation (r)	ICC
Tb.vBMD (g/cc)	1.16 ± 0.03	1.15 ± 0.03	0.95	0.93
Tb.NA (1/mm)	0.64 ± 0.15	0.80 ± 0.17	0.87	0.58
Tb.PW (*μ*m)	1206.97 ± 157.67	1297.02 ± 112.90	0.96	0.75
Tb.Th (*μ*m)	306.03 ± 44.52	237.27 ± 35.08	0.99	0.39
Tb.Sp (*μ*m)	400.64 ± 85.52	366.36 ± 95.16	0.92	0.85

**Figure 3.**
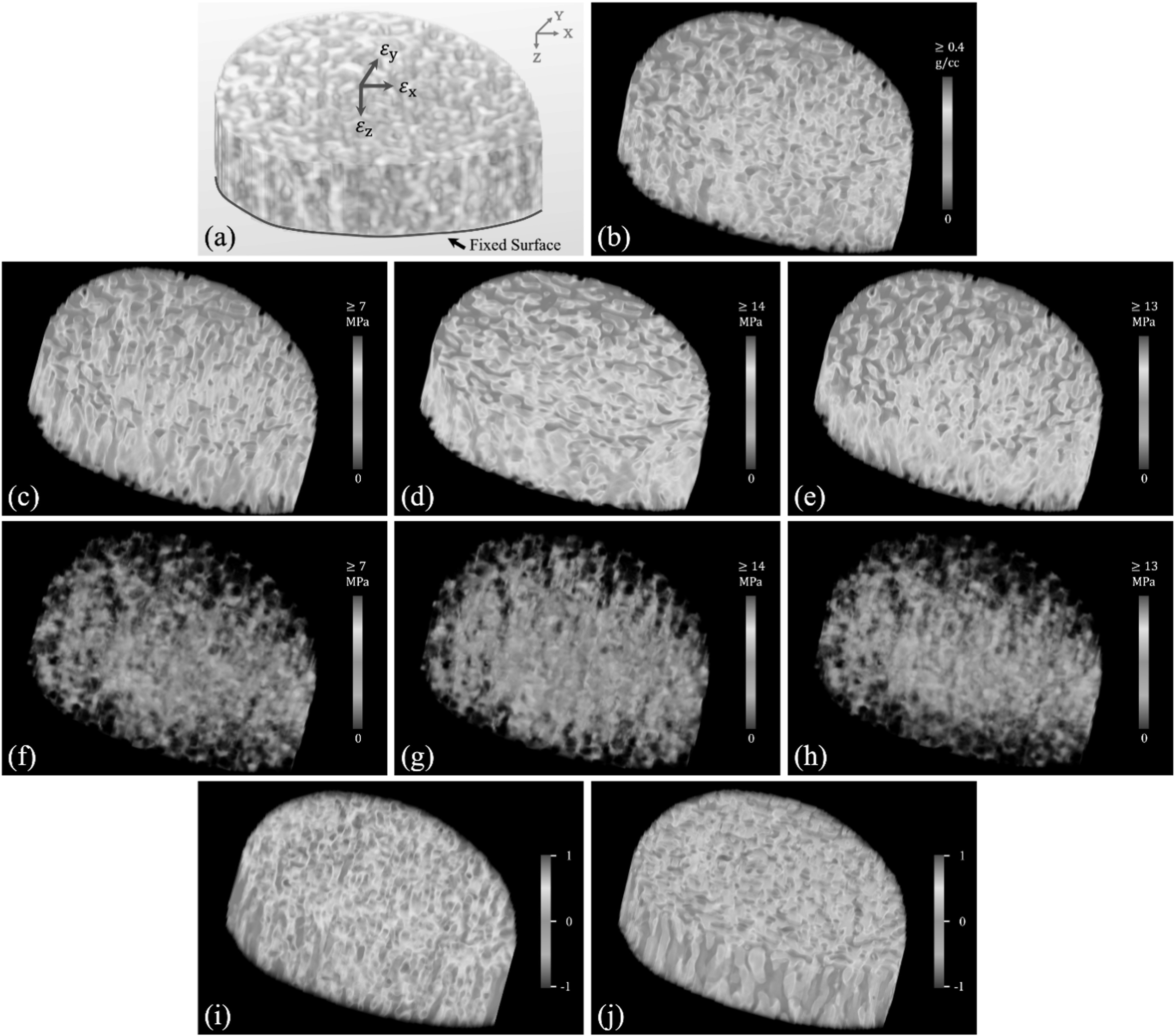
Results of FEA on LR CT under different loading conditions. (a), (b) Illustration of boundary conditions and loading setup for nonlinear FEA (a) and the color-coded volume rendition of CHA density map (b) on a target VOI. (c)–(e) Stress distribution under compressive (c), x-shear (d), and y-shear (e) loading conditions. (f)–(g) Stress leakages over the marrow space for compressive (f), x-shear (g), and y-shear (h) loading. For each loading condition, the overall stress distribution and the stress leakage in marrow space are displayed using the same color-scale. (i,j) Normalized stress differences between compressive and x-shear (i) and between compressive and y-shear (j) loading conditions. Regions appearing in red (or, blue) indicate voxels with higher (respectively, lower) compressive stress compared to shear stress, while region appearing in green, yellow, and cyan represent voxels with similar compressive and shear stresses.

Figure 3 presents the results of application of FEA on LR CT. A volume rendition of the target VOI with the applied loading and boundary conditions for FEA is shown in (a). A color-coded rendition of the CHA density map of (a) is shown in (b). Figures (c)–(e) show the renditions of observed von Mises stress over the VOI for compressive, x- and y-shear loading conditions, respectively. Figures (f)–(h) show the stress distribution over the marrow region at the respective loading conditions. Agreements in high stress lines (red) in (c)–(e) with Tb microstructure (red) in (b) are visible, and low stress values (blue) in (f)–(h) over the marrow region confirm nominal stress leakage under all loading conditions. To examine the difference in loading patterns between compressive and shear loading conditions, voxel-wise differences between normalized stresses under compressive and each of the two shear loading conditions were computed and the difference maps are rendered in (i, j). For a given image and a specific loading condition, von Mises stress values were normalized using a linear mapping function, where the von Mises stresses at and above the 99th percentile were mapped to ‘1’. Thus, in both stress difference images, the values lie in the [−1 1] interval with positive values indicating higher compressive stress. For quantitative analysis, BMD values were mapped to a normalized measure of bone volume fraction (BVF) using a previously validated method (Chen *et al* 2018). The fraction of loaded bone voxels in a given image under a specific loading condition was computed as $\displaystyle {\sum }_{p\in B}\tau \left(p\right)BVF\left(p\right)/\displaystyle {\sum }_{p\in B}BVF\left(p\right),$ where *B* is the set of Tb voxels and $\tau \left(\cdot \right)$ and $BVF\left(\cdot \right)$ are the normalized stress and BVF values at individual voxels. The mean±std of the fraction of loaded Tb voxels for compressive, x-shear, and y-shear loading conditions in LR CT images were 0.69 ± 0.06, 0.65 ± 0.09, 0.53 ± 0.08, respectively. Paired t-test between the fraction of loaded Tb voxels for compressive loading and that for each of the two shear loading showed that significantly greater fractions (p < 0.05) of Tb voxels were loaded in compressive loading as compared to x- and y-shear loadings, which is consistent with the stress difference renditions of figures 3(i), (j). Similar results were observed for HR CT images.

**Figure 4.**
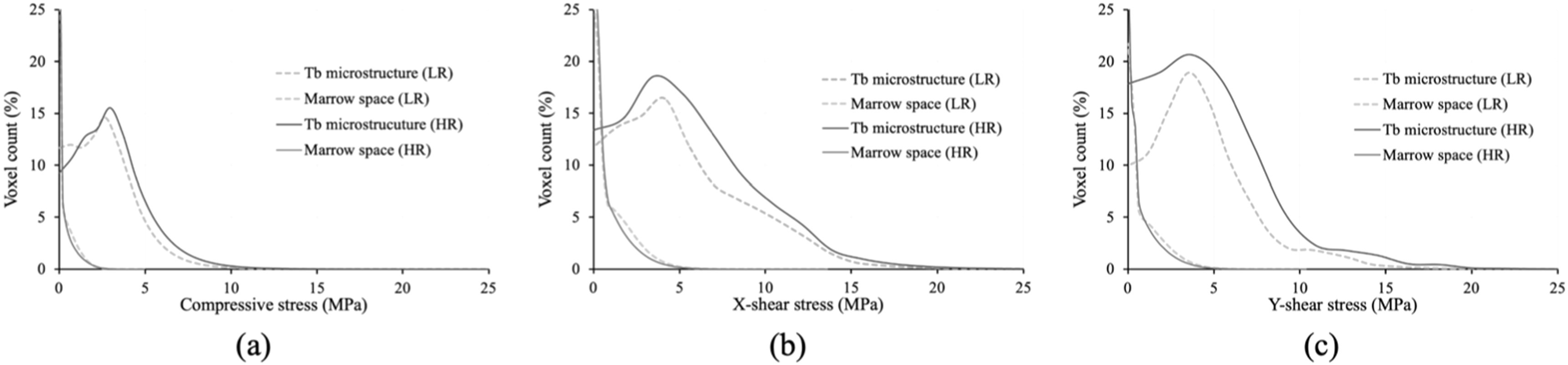
Histograms of Tb and marrow voxel counts at different stress values for LR and HR CT scans under compressive (a), x-shear (b), and y-shear (c) loading conditions.

The normalized stress histograms computed over Tb and marrow regions of all LR (n = 20) and HR (n = 20) scans under different loading conditions are separately shown in figure 4. For all loading conditions, the stress histograms computed over marrow region (green) fall rapidly and mean±std of the absorbed stress were 0.4 ± 0.5, 1.1 ± 1.1, and 1.0 ± 1.0 MPa for LR scans at compressive and x- and y-shear loading, respectively, and 0.4 ± 0.5, 0.9 ± 1.0, and 0.9 ± 0.9 MPa for HR scans at respective loading conditions. On the other hand, for both LR and HR scans the modes of the histograms computed over the Tb region (red) were higher than the average stress values of marrow regions under the same loading condition. Mean±std of the stress supported by Tb voxels for compressive and x- and y-shear loading scans were 2.3 ± 1.7, 4.1 ± 3.2, and 4.3 ± 2.9 MPa, respectively, for LR scans and 2.7 ± 1.9, 4.9 ± 3.5, and 5.1 ± 3.2 MPa, respectively, for HR scans. The difference in stress distribution over Tb and marrow regions was also significant (p < 10^−12^) for LR as well as HR scans at every loading condition. These observations are in line with the fundamental principle of structural mechanics at *in vivo* CT imaging stating that the majority of stress is absorbed by Tb compared to marrow space. It was observed that, at a matching loading condition, the area under the curve of the HR Tb stress histogram was higher than that of the LR Tb stress histogram. This observation reasserts that greater volume of Tb network was detected in HR scans as compared to LR scans.

A limitation of the current study is that the study subjects were young adult and generally-healthy to serve the purpose of the Iowa Bone Development Study (Janz *et al* 2002, Janz *et al* 2005) and the findings may not be generalizable to elderly populations with weaker Tb and reduced Tb network area density (Ding and Hvid 2000, Khosla *et al* 2006). Toward an effort to address this issue, we identified the Tb region in CT scans of the young adult population that represents the Tb network density in an elderly population at the 4%–6% tibial site. Specifically, we selected subjects with preserved lung function (n = 32, 12 Male, mean ± std of age: 64.0 ± 4.7 years) from the elderly samples of an Iowa cohort of the ongoing COPDGene Study (Regan *et al* 2011), who underwent HR ankle CT scans using the Siemens FORCE CT imaging protocol (see section 2.1). The optimum 2% tibial site in CT scans of the current study subjects representing the 4%–6% tibial site in the elderly population was determined by minimizing the Mahalanobis distance (De Maesschalck *et al* 2000) of Tb.NA distributions derived from the target 4%–6% tibial site in the elderly subjects and the candidate 2% tibial site in the current study subjects; the optimum 2% tibial site was experimentally observed at 6.2%–8.2% distal tibial location. All experiments were repeated for the simulated ‘elderly VOI,’ and high inter-scanner reproducibility was observed, with ICC values of 0.98, 0.95, and 0.94 for compressive, and X- and Y-shear moduli, respectively. The observed ICC value for the microstructural measure Tb network area density (Tb.NA) was 0.69. Thus, the simulated experiment suggests that the CT-based nonlinear FEA measures in an elderly population may be generalizable across scanners with different resolution features.

## 4. Conclusion

This paper presents and evaluates a nonlinear continuum FE method for computation of Tb microstructural strength from *in vivo* CT imaging that does not require binary separation of bone and marrow regions, while accounting for bone micro-distribution at the level of individual trabeculae. High reproducibility of modulus measures was observed experimentally under different loading conditions for young adult as well as simulated elderly Tb VOIs. Also, it was found that stress micro-propagations were primarily confined within the Tb micro-network, with minimal leakages over the marrow space, complying with the basic principle of structural mechanics at the *in vivo* CT resolution. Overall, our results demonstrate that *in vivo* CT-based FEA is a viable method for assessment of human bone microstructural strength, and high agreement of strength measures from low- and high-resolution CT scanners justifies its generalizability in multi-site and longitudinal human studies that often must use different scanners over the study period.

## Figures and Tables

**Figure A1.**
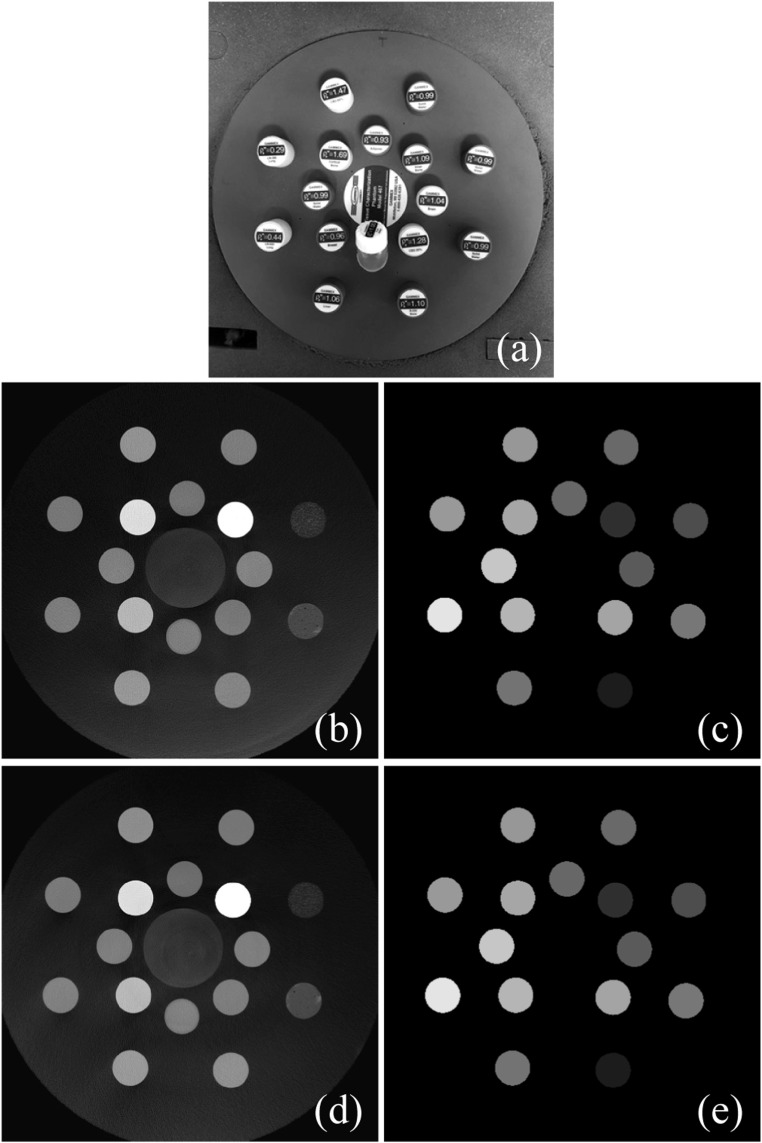
CT-to-BMD calibration phantom and scans. (a) A photograph of the Gammex RMI *4*6*7* Tissue Characterization Phantom used for CT-to-BMD calibration. (b), (c) An axial image slice from the low-resolution scan of the Gammex phantom on a Siemens FLASH scanner (b) and segmented cylinders for different tissue characterization (c). (d), (e) Same as (b), (c) but for the high-resolution Siemens FORCE scanner.

**Figure A2.**
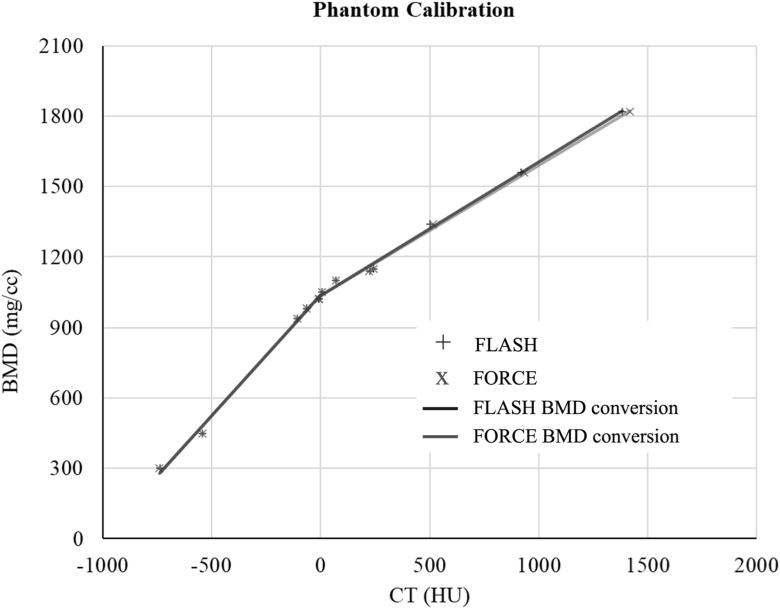
CT-to-BMD calibration curve for the low-resolution FLASH and high-resolution FORCE scanners.

## Data Availability

The data cannot be made publicly available upon publication because they contain sensitive personal information. The data that support the findings of this study are available upon reasonable request from the authors.

## APPENDIX : CT-to-bone mineral density (BMD) calibration

Separate CT-to-Bone mineral density (BMD) calibration curves were derived for low- and high-resolution scanners using a Gammex RMI 467 Tissue Characterization Phantom (Gammex RMI, Middleton, WI, USA), which includes sixteen cylinders with known physical densities (mg/cc) (figure A1(a)). Specifically, for each scanner, the Gammex phantom was scanned using the matching CT imaging and reconstruction protocol using for human scans; axial image slices of LR and HR scans of the phantom are shown in figures A1(b), (d). Each of the sixteen cylinders was automatically located, segmented, and labelled in a phantom scan using an automated method developed in our laboratory based on thresholding, connectivity, morphological analysis (figures A1(c), (e)). To eliminate variabilities in CT intensity values due to partial voluming each segmented cylindrical volume was eroded by five voxels, and the average CT value over the eroded region was computed. For each scanner, the average CT intensity values and vendor-provided physical densities of individual cylinders were recorded on a CT-to-physical density plot (figure A2). Finally, the CT-to-BMD calibration curve was computed for each scanner from the corresponding CT-to-physical density plot by fitting a linear equation to the data points of cylinders with physical density less than water density and another linear equation to the data points of remaining cylinders (figure A2).

## References

[bpexacbb0abib1] Bauer J S, Sidorenko I, Mueller D, Baum T, Issever A S, Eckstein F, Rummeny E J, Link T M, Raeth C W (2014). Prediction of bone strength by *μ*CT and MDCT-based finite-element-models: How much spatial resolution is needed?. Eur. J. Radiol..

[bpexacbb0abib2] Boutroy S, Van Rietbergen B, Sornay‐Rendu E, Munoz F, Bouxsein M L, Delmas P D (2008). Finite element analysis based on *in vivo* HR‐pQCT images of the distal radius is associated with wrist fracture in postmenopausal women. J. Bone Miner. Res..

[bpexacbb0abib3] Bouxsein M L, Boyd S K, Christiansen B A, Guldberg R E, Jepsen K J, Muller R (2010). Guidelines for assessment of bone microstructure in rodents using micro-computed tomography. J. Bone Miner. Res..

[bpexacbb0abib4] Chen C, Jin D, Zhang X, Levy S M, Saha P K (2016). Segmentation of trabecular bone for *in vivo* CT imaging using a novel approach of computing spatial variation in bone and marrow intensities.

[bpexacbb0abib5] Chen C, Zhang X, Guo J, Jin D, Letuchy E M, Burns T L, Levy S M, Hoffman E A, Saha P K (2018). Quantitative imaging of peripheral trabecular bone microarchitecture using MDCT. Med. Phys..

[bpexacbb0abib6] Chevalier Y, Pahr D, Allmer H, Charlebois M, Zysset P (2007). Validation of a voxel-based FE method for prediction of the uniaxial apparent modulus of human trabecular bone using macroscopic mechanical tests and nanoindentation. J. Biomech..

[bpexacbb0abib7] De Maesschalck R, Jouan-Rimbaud D, Massart D L (2000). The mahalanobis distance. Chemometr. Intell. Lab. Syst..

[bpexacbb0abib8] Ding M, Hvid I (2000). Quantification of age-related changes in the structure model type and trabecular thickness of human tibial cancellous bone. Bone.

[bpexacbb0abib9] Guha I, Zhang X, Rajapakse C S, Chang G, Saha P K (2022). Finite element analysis of trabecular bone microstructure using CT imaging and continuum mechanical modelling. Med. Phys..

[bpexacbb0abib10] Jang I, Bae G, Song J, Kim H, Park N (2020). Fracture envelopes on the 3D-DIC and hybrid inverse methods considering loading history. Mater. Des..

[bpexacbb0abib11] Janz K F, Burns T L, Levy S M (2005). Tracking of activity and sedentary behaviors in childhood: the Iowa bone development study. American journal of preventive medicine.

[bpexacbb0abib12] Janz K F, Levy S M, Burns T L, Torner J C, Willing M C, Warren J J (2002). Fatness, physical activity, and television viewing in children during the adiposity rebound period: the Iowa bone development study. Preventive medicine.

[bpexacbb0abib13] Keyak J, Lee I, Nath D, Skinner H (1996). Postfailure compressive behavior of tibial trabecular bone in three anatomic directions. J. Biomed. Mater. Res..

[bpexacbb0abib14] Keyak J, Sigurdsson S, Karlsdottir G, Oskarsdottir D, Sigmarsdottir A, Kornak J, Harris T, Sigurdsson G, Jonsson B, Siggeirsdottir K (2013). Effect of finite element model loading condition on fracture risk assessment in men and women: the AGES-Reykjavik study. Bone.

[bpexacbb0abib15] Keyak J H, Kaneko T S, Tehranzadeh J, Skinner H B (2005). Predicting proximal femoral strength using structural engineering models’. Clin. Orthop. Relat. Res..

[bpexacbb0abib16] Khosla S, Riggs B L, Atkinson E J, Oberg A L, McDaniel L J, Holets M, Peterson J M, Melton L J (2006). Effects of sex and age on bone microstructure at the ultradistal radius: a population-based noninvasive *in vivo* assessment. J. Bone Miner. Res..

[bpexacbb0abib17] Kleerekoper M, Villanueva A R, Stanciu J, Rao D S, Parfitt A M (1985). The role of three-dimensional trabecular microstructure in the pathogenesis of vertebral compression fractures. Calcified Tissue Int..

[bpexacbb0abib18] Legrand E, Chappard D, Pascaretti C, Duquenne M, Krebs S, Rohmer V, Basle M F, Audran M (2000). Trabecular bone microarchitecture, bone mineral density, and vertebral fractures in male osteoporosis. J. Bone Miner. Res..

[bpexacbb0abib19] Li C, Jin D, Chen C, Letuchy E M, Janz K F, Burns T L, Torner J C, Levy S M, Saha P K (2015). Automated cortical bone segmentation for multirow-detector CT imaging with validation and application to human studies. Med. Phys..

[bpexacbb0abib20] Liu X S, Zhang X H, Rajapakse C S, Wald M J, Magland J, Sekhon K K, Adam M F, Sajda P, Wehrli F W, Guo X E (2010). Accuracy of high‐resolution *in vivo* micro magnetic resonance imaging for measurements of microstructural and mechanical properties of human distal tibial bone. J. Bone Miner. Res..

[bpexacbb0abib21] Liu Y, Jin D, Li C, Janz K F, Burns T L, Torner J C, Levy S M, Saha P K (2014). A robust algorithm for thickness computation at low resolution and its application to *in vivo* trabecular bone CT imaging. IEEE Trans. Biomed. Eng..

[bpexacbb0abib22] Logan D L (2011). A first course in the finite element method.

[bpexacbb0abib23] MacNeil J A, Boyd S K (2007). Accuracy of high-resolution peripheral quantitative computed tomography for measurement of bone quality. Med. Eng. Phys..

[bpexacbb0abib24] Meijering E H, Niessen W J, Pluim J P, Viergever M A (1999). Quantitative comparison of sinc-approximating kernels for medical image interpolation, translated by Springer.

[bpexacbb0abib25] National Osteoporosis Foundation (2017). https://cdn.nof.org/wp-content/uploads/2017_NOF_Annual_report_v6_final.pdf.

[bpexacbb0abib26] Nishiyama K, Macdonald H, Hanley D, Boyd S (2013). Women with previous fragility fractures can be classified based on bone microarchitecture and finite element analysis measured with HR-pQCT. Osteoporosis international.

[bpexacbb0abib27] Parfitt A M, Mathews C H E, Villanueva A R, Kleerekoper M, Frame B, Rao D S (1983). Relationships between surface, volume, and thickness of iliac trabecular bone in aging and in osteoporosis - implications for the microanatomic and cellular mechanisms of bone loss. J. Clin. Invest..

[bpexacbb0abib28] Rajapakse C S, Magland J F, Wald M J, Liu X S, Zhang X H, Guo X E, Wehrli F W (2010). Computational biomechanics of the distal tibia from high-resolution MR and micro-CT images. Bone.

[bpexacbb0abib29] Rayudu N M, Anitha D P, Mei K, Zoffl F, Kopp F K, Sollmann N, Löffler M T, Kirschke J S, Noël P B, Subburaj K (2020). Low-dose and sparse sampling MDCT-based femoral bone strength prediction using finite element analysis. Archives of Osteoporosis.

[bpexacbb0abib30] Regan E A, Hokanson J E, Murphy J R, Make B, Lynch D A, Beaty T H, Curran-Everett D, Silverman E K, Crapo J D (2011). Genetic epidemiology of COPD (COPDGene) study design. COPD: Journal of Chronic Obstructive Pulmonary Disease.

[bpexacbb0abib31] Van Ruijven L, Mulder L, Van Eijden T (2007). Variations in mineralization affect the stress and strain distributions in cortical and trabecular bone. J. Biomech..

[bpexacbb0abib32] Saha P K, Xu Y, Duan H, Heiner A, Liang G (2010). Volumetric topological analysis: a novel approach for trabecular bone classification on the continuum between plates and rods. IEEE Trans. Med. Imaging.

[bpexacbb0abib33] Seeman E, Delmas P D (2006). Bone quality--the material and structural basis of bone strength and fragility. New Engl. J. Med..

[bpexacbb0abib34] Ulrich D, van Rietbergen B, Weinans H, Rüegsegger P (1998). Finite element analysis of trabecular bone structure: a comparison of image-based meshing techniques. J. Biomech..

[bpexacbb0abib35] Watts N B, Bilezikian J P, Camacho P M, Greenspan S L, Harris S T, Hodgson S F, Kleerekoper M, Luckey M M, McClung M R, Pollack R P (2010). American association of clinical endocrinologists medical guidelines for clinical practice for the diagnosis and treatment of postmenopausal osteoporosis. Endocrine practice.

[bpexacbb0abib36] Wehrli F W, Saha P K, Gomberg B R, Song H K, Snyder P J, Benito M, Wright A, Weening R (2002). Role of magnetic resonance for assessing structure and function of trabecular bone. Topics in Magnetic Resonance Imaging.

[bpexacbb0abib37] Zhang N, Magland J F, Rajapakse C S, Bhagat Y A, Wehrli F W (2013). Potential of *in vivo* MRI‐based nonlinear finite‐element analysis for the assessment of trabecular bone post‐yield properties. Med. Phys..

